# Learning Through Doing: Comprehensive Programming for a Training Program in Cancer Disparities

**DOI:** 10.1007/s13187-022-02235-y

**Published:** 2022-12-10

**Authors:** Kayce D. Solari Williams, Kamisha Hamilton Escoto, Crystal Roberson, Kathy Le, Lorraine R. Reitzel, Lorna H. McNeill, Shine Chang

**Affiliations:** 1 Department of Psychological, Health, & Learning Sciences, University of Houston, Houston, TX USA; 2 George R. Brown School of Engineering–Active Engineering Communication Program, Houston, TX USA; 3 Department of Health Disparities Research, The University of Texas MD Anderson Cancer Center, Houston, TX USA; 4 School of Medicine, University of Texas Health Science Center at San Antonio, San Antonio, TX USA; 5 Department of Epidemiology, Cancer Prevention Research Training Program, The University of Texas MD Anderson Cancer Center, Houston, TX USA

**Keywords:** Cancer prevention research training, Disparities, Research workforce, Graduate education, Educational approaches

## Abstract

In the United States, preparing researchers and practitioners for careers in cancer requires multiple components for success. In this reflection article, we discuss our approach to designing a comprehensive research training program in cancer disparities. We focused on elements that provide students and early career scientists a deep understanding of disparities through first-hand experiences and skills training necessary to build a research career in the area. Our Educational Program sits within the framework of an NCI P20 program, “UHAND (U﻿niversity of Houston/MD And﻿erson Cancer Center)”, jointly established by an NCI-designated comprehensive cancer center and a minority-serving university as a collaborative partnership devoted to the elimination of cancer inequities among disproportionately affected racial and ethnic groups (UHAND Program to Reduce Cancer Disparities; NCI P20CA221696/ P20CA221697). The Education Program was designed to build on and enhance skills that are critical to pursuing a career in cancer disparities research at the undergraduate, doctoral, and post-doctoral levels—such as scientific communication, career planning and development, professional and community-based collaboration, and resilience in addition to solid scientific training. As such, our program integrates (1) opportunities for learning through service to community organizations providing resources to populations with documented cancer disparities, (2) a tailored curriculum of learning activities with program leadership and mentored research with scientists focused on cancer disparities and cancer prevention, (3) professional development training critical to career success in disparities research, and (4) support to address unique challenges faced by trainees from backgrounds that are historically underrepresented in research.

## Introduction

What knowledge and skills are essential for workforce training to advance research in cancer disparities, particularly for racial/ethnic minority students and trainees? What experiences prepare students to define cutting edge research questions and develop innovative yet scientifically sound methods to address them? Beyond research training, how is self-awareness fostered for career development so that racial/ethnic minority students are prepared to be leaders in their chosen fields? These were the questions we asked ourselves as we developed the Education Program for our *National Cancer Institute Center for Research to Reduce Cancer Health Disparities* (NCI CRCHD) “Feasibility Studies to Build Collaborative Partnerships in Cancer Research” P20 award, as their careful consideration are imperative to improving racial/ethnic diversity in the health science workforce. Black/African American (hereafter Black), Hispanic/Latinx (hereafter Hispanic), and Native Americans are underrepresented throughout the academic pipeline, holding less than 10% of academic doctoral positions at universities or 4-year colleges in science, engineering, and health [1]. Diversifying the research workforce is further challenged as the proportion of individuals from underrepresented racial/ethnic minority groups earning doctoral degrees remains low; for example, in 2018 racial/ethnic minority students received just 13.6% of all doctoral degrees awarded in science and engineering (SE) [1]. Further, while female minority students have higher enrollment in SE doctoral programs than their minority male counterparts, only 4.6% of SE doctoral students are Black women and 7.5% are Hispanic women [1]. Thus, the numbers of underrepresented scientists-in-training are small and cannot at this pace address the workforce needs to eliminate cancer disparities [1]. The NCI P20 grant mechanism (PAR-16–084) was designed to create partnerships between cancer centers and institutions serving underrepresented students and health disparity populations to build training programs to address these disparities.

Our program, UHAND (U﻿niversity of H﻿ouston/MD And﻿erson Cancer Center), is a collaboration between University of Houston (UH) and the University of Texas MD Anderson Cancer Center (MD Anderson)—institutions devoted to training the next generation of minority and women scientists in eliminating cancer inequities through the reduction of social and physical risk factors among minority racial/ethnic populations [2]. As the nation’s fourth largest city and one of the most racially and ethnically diverse, the city of Houston served as an ideal setting for this partnership [3, 4]. The Houston metropolitan statistical area, a 10-county area that includes Harris County, is the largest in Texas. In 2020, Houston had a population of more than 2.3 million, with 50% speaking a language other than English at home, 23% identifying as Black, and 45% as Hispanic [4]. The city has wide social and economic disparities, with more than 25% of those under 65 being uninsured and 20% living below the poverty level [4]. Regarding cancer disparities, from 2015 to 2019 Black residents of Harris County had cancer mortality rates that were 28% higher than the overall county average [5]. Clearly, innovations are needed to address these disparities, readily positioning our partnership to craft a program that tackled clear, real-world issues related to cancer disparities while leveraging abundant resources in both the academic and racial/ethnic communities.

University of Houston (UH) is the leading public research university in Houston and has the second most ethnically diverse student population at a major research university in the U.S. [6]. As a designated Hispanic-serving institution and Asian American and Native American Pacific Islander-serving institution, UH served as a conduit to underrepresented students and faculty for recruitment into the program and brought expertise in how to recruit and retain students from these populations. MD Anderson, located in the Texas Medical Center, is one of the world’s largest and most respected NCI-designated comprehensive cancer centers devoted exclusively to cancer patient care, research, education, and prevention. Both UH and MD Anderson have a strong commitment to health equity and working with community partners to improve health outcomes. Our intention was to combine the strengths of both institutions to create a program that would serve undergraduate, graduate, and post-doctoral students (hereafter referred to as UHAND scholars; this article focuses on programming for undergraduate and graduate students only) and prepare them for the next step in a cancer disparities career.

## UHAND Education Program (EP)

The UHAND Program had three main components: a Pilot Research Program that provided support to new investigators in developing innovative cancer disparities research, a Community Outreach Program that provided structured programming and experiences to increase scholars’ skills in working with communities and community-engaged research, and an Education Program (EP) to train scholars on the attitudes, knowledge, and skills required to conduct cancer disparities research (full program details are published elsewhere) [2]. Our focus in this reflection piece is the EP. The EP was led by one Director from each partner institution (SC, KDSW) with additional leadership from MD Anderson (KHE). Each member of this leadership team worked with the UHAND PIs and staff in developing the EP and assumed responsibility for leading specific components.

EP components included (1) mentored research projects [2], (2) opportunities to participate in interactive seminars with leaders and seasoned researchers in the fields of cancer disparities and cancer prevention (SC, KHE), (3) service learning experiences with community organizations that provide services to populations with documented cancer disparities (KDSW), (4) hands-on professional development workshops that provide essential skills for a career in disparities research (SC, KHE, KDSW), and (5) supportive resources to address unique challenges faced by students from backgrounds that are historically underrepresented in research (SC, KDSW). Key to the EP were mentored research experiences that focused on disciplinary scientific training in cancer disparities. Scholars were paired with faculty research mentors at UH or MD Anderson who had expertise in cancer risk, social determinants of health, and clinical and population cancer research in Black and Hispanic populations. In addition to increasing scholar knowledge of cancer disparities and research design, the EP’s training objectives focused on imparting critical skills (e.g., communication and interpersonal skills and resiliency) necessary to empower minority scientists to thrive in academic medicine. Given that the EP’s leadership included education specialists with experience as training program directors, the EP aimed to build on the mentored research experience and other UHAND core program elements to develop a well-rounded program prioritizing both personal and professional development. Training competencies that guided the EP, adapted from the literature, are outlined in Table 1 [7, 8].

**Table 1:** Competencies in training cancer disparities scientists targeted in the UHAND Educational Program

Competency
Attitudes
• Values collaboration with and in diverse communities
• Empathizes with populations experiencing disparities due to race or ethnicity, disability, religion, culture, and sexual orientation
• Committed to reducing cancer disparities through social change
Knowledge
• Understands cancer risk factors (e.g., tobacco use, diet, and physical activity) and cancer disparities in black/AAs and Hispanics/Latinx adults
• Understands social determinants of health
• Knows the principles of community-based participatory research
• Understands research ethics and responsible conduct of research
• Knows core theories and research methods used in biological, behavioral, and population sciences
Skills
• Communication and interpersonal skills
• Persistence in overcoming obstacles to community-engaged research
• Shared decision-making
• Community-based teaching and learning approaches
• Ability to work with communities in conjunction with advocacy groups
• Resilience in navigating the systemic barriers experienced by minority faculty and students (e.g., discrimination, microaggressions, and sexism)

### Interactive Seminars

We created a new Cancer Disparities seminar series, specifically for the UHAND scholars, to orient them to cutting edge research in cancer disparities and foster opportunities to meet and interact with faculty experts in a small group setting of peers. Faculty experts from both UH and MD Anderson delivered interactive lectures on topics ranging from social determinants of health and disparities in clinical trial participation to shared decision-making in cancer. The seminars were meant to broaden the scholars’ knowledge of cancer disparities and showcase the range of research topics and approaches they could explore in cancer disparities. We also wanted to empower scholars to be comfortable using their voice, asking questions, and interacting with faculty and peers. We assigned each scholar to introduce a speaker and facilitate discussion after the faculty presentation; EP leadership delivered training sessions on these skills at the start of the series. UHAND scholars also attended weekly *Cancer Prevention and Control Grand Rounds (Grand Rounds)* at MD Anderson, which featured the research and practice of prominent leaders nationwide in cancer prevention research and control. Following Grand Rounds, the scholars were invited to attend a trainee-led lunch session with speakers to learn more about their career trajectory and career mentoring, ask additional questions about their research, and discuss shared research interests.

### Service Learning

In addition to their interactions with research experts, scholars engaged in service learning experiences in a variety of community settings. Beyond the fundamental intent of service learning (combining academic preparation with volunteer opportunities), we intended for scholars’ direct engagement in community service activities to expand their understanding of relevant issues and increase active participation in their communities of interest [9]. UHAND scholars were charged with establishing meaningful connections with community partners through engagement in small projects. For example, we had placements at cancer advocacy organizations (scholars assisted with intake questionnaires, community interviews, and led community cancer support groups), cancer coalitions (scholars helped develop an outcomes evaluation plan), and non-profits supporting health lifestyles (scholars taught nutrition classes to underserved community members). The experiences were designed to help scholars integrate didactic information with real-life and tangible experiences while allowing them to enhance their social and advocacy skills, cultivate civic and ethical responsibility, and inform their career exploration and planning. EP leadership advised the scholars to serve with intention and to focus on learning and understanding how to work with communities as they advanced their own research and service career paths. At the conclusion of the program, UHAND EP leadership and scholars discussed the experience, giving scholars the opportunity to self-reflect on their projects in the context of serving both the community and the community organizations. As the program matured, the service learning experience was folded into the Community Outreach Program, which was expanded to include elements such as community shadowing experiences, community mentoring, and a lecture series featuring area community-based leaders in cancer and health disparities.

### Hands-on Professional Development

We created professional development opportunities that leveraged both existing training infrastructure and EP leadership expertise to build key skills in career management and scientific communication [10]. In our experience, it is important that scholars are familiar with and have experience using self-assessment tools and resources that confirm they are pursuing training programs that connect to their emerging professional identities. The MD Anderson EP Director leads an established career development workshop adapted from the book “What Color is Your Parachute?” designed to guide participants in identifying their values, interests, and transferrable skills to map onto potential career paths [11]. To provide further career perspective, scholars attended weekly “Career Conversations in Cancer Prevention” sessions each summer, organized by existing training programs at MD Anderson [8]. Designed for students to learn firsthand about career opportunities in cancer prevention and cancer disparities, UHAND scholars met with oncologists, clinical faculty, researchers, graduate students, and community members engaged in cancer prevention along with other trainees from programs across MD Anderson. Here, speakers candidly shared their career journeys, including how they became interested in and trained for cancer prevention, their current work, the populations they serve, and obstacles they have overcome.

Additionally, we prioritized programming that would allow students to strengthen skills and increase confidence using different forms of scientific communication. Customized workshops in both oral and written communication were offered, given the importance of such skills for retaining graduate and postgraduate students in research careers [12]. For example, the scholars participated in the “Scientific Elevator Speech” training, a long-standing workshop integrated in MD Anderson’s cancer prevention training programs [8]. The workshop was designed to help trainees communicate research to a general audience clearly and concisely. Scholars learned strategies to present themselves, their research question, research findings, and impact of their work engagingly in 90 s. Further, EP leadership from both UH and MD Anderson conducted writing workshops to help students develop a writing practice, improve their skills, and form writing communities. Modeled from the book “Becoming an Academic Writer,” scholars participated in writing exercises, learned strategies to increase their writing productivity, and provided peer feedback on works in progress [13]. Finally, based on feedback from the scholars and mentors, we added training sessions on conflict resolution and time management, utilizing experts from both UH and MD Anderson. A curated list of other professional development activities available at both institutions were also communicated weekly to scholars via email.

### Supportive Resources

The EP leadership were intentional in creating a training environment reducing common challenges racial/ethnic minority students experience [14]. We aimed to create a community of scholars to reduce feelings of social isolation, offer support and supplemental mentorship to ensure trainee professional and personal well-being, and serve as a ‘safe space’ for scholars as they navigated both the academic and wider environment. For proximity and ease of access, the UH EP Director offered weekly office hours so the scholars could visit and connect both formally and informally. Scholars were encouraged to ask questions about career paths, writing, personal and professional growth opportunities, and/or anything related to the program. Additionally, EP leadership conducted regular check-ins and exit interviews to allow more time for feedback and discussion of issues such as scholar needs, satisfaction with the program, and confidence in moving forward in their career path. This consistent presence ultimately provided an additional avenue for mentoring, with focus on what was most important to scholars in their academic journey at the moment. Further, we organized several social activities for the scholars to connect and feel like they were part of a scholarship community. Social activities such as holiday parties, bowling outings, and end of semester socials provided opportunities for interaction beyond the formal programming. The “graduation ceremony” (conducted virtually because of the pandemic) was the culminating event of the UHAND program for each of two scholar cohorts. The ceremony was attended by UHAND team members, advisory committee members, mentors, friends, and family and included a video diary of cohort activities, a keynote speech by a UHAND PI, and a spotlight moment focused on each graduating scholar. We concluded with a “Where are they now?” update on alumni scholars. Overall, EP leadership observed well-rounded growth in all scholars by the end of their program.

## Summary of Reflections

As we implemented the UHAND EP program, we continually asked ourselves the questions posed at the start of this reflection article. We felt strongly that purposeful investment of time and resources on holistic trainee programming would be critical to diversifying the scientific workforce. Because discriminatory practices regarding race and gender, including unconscious bias, exist in health and science disciplines [15, 16], training programs like UHAND are critical for helping trainees strengthen resilience and career persistence skills because they intentionally establish inclusive skill-building curricula and situate them in inclusive learning environments; thus, arming individuals to navigate career paths and fostering learning environments free of bias are both needed to increase diversity in the health and science workforces. With support and input from the UHAND team, the EP leadership created a curriculum of activities for the UHAND scholars to provide well-rounded opportunities for scientific, professional, and importantly, social development. The curriculum—its structure, activities, staff, and mentors—was well received by the scholars as confirmed by objective assessments published elsewhere and our informal discussions (e.g., check-ins with scholars) [2]. Program strengths noted by scholars included the extensive support network in the Program (e.g., UHAND and EP leadership, faculty, and staff) opportunities to learn from and interact with diverse faculty in smaller, less formal settings (e.g., disparities seminars); career development workshops; and opportunities to build community with their fellow scholars. We noticed strong bonds with other UHAND scholars and understood some scholars looked to their UHAND colleagues as both friends and role models. We learned that scholars felt the time spent with a wide range of professionals during seminars was valuable and reassuring, particularly when presenters were receptive and responded openly to questions and inquiries about career paths. We believe these program features—fostered by UHAND leadership presence, availability, and commitment—distinguish our EP program and should serve as an exemplar model in the field.

When considering the powerful roles and impact of communication and trust for students during implementation of a wholistic training curriculum, we recognize the need for consistent and active messaging and participation from leadership, support staff, and scholars. Establishing and maintaining an open communication style of and trust used by program leadership were rooted in the aim to diversify the field. For example, we openly encouraged scholars’ presentation of their authentic selves throughout the experience. We reinforced this value by beginning our interactions with questions about the scholars’ personal well-being and that of their families, actively listening and expressing empathy as they described struggling during the pandemic both with academics and health. If a scholar was ever uncertain about how to approach a task, had a scheduling conflict, or was unclear about their career trajectory, discussions were forthcoming on both ends in that we offered multiple approaches but also helped scholars practice using their problem-solving skills. EP leadership expressed gratitude with the scholars’ willingness to share openly and the scholars’ reciprocated the sentiment. The UHAND experience is an example of providing an environment that is structured and rigorous while fostering growth mindset in trainees that benefits every stakeholder.

Training programs often focus primarily on concrete or quantifiable research outcomes as a measure of professional aptitude or success. While this may be key to advancing science and knowledge, we also recognize a wider scope of factors integral to the scientific and professional success of individuals, particularly considering the broader working environment in which we work and the complexity of problems facing health and science today. The UHAND Program was established during historically challenging times—during the COVID-19 pandemic as well as extreme racial, political, and social unrest in the nation. Often occurring on a national or international level that may not overtly reach individual trainees, our view is that training programs have responsibility to acknowledge and respond to these issues, helping young adults process information and emotions as both relevant to their interest in addressing health disparities and in their development as professionals in the field. In response to #ShutDownSTEM (initiative from an intersectional coalition of STEM professionals and academics taking action for Black lives; shutdownstem.com/about), for example, EP leaders changed the topic of a scheduled seminar to have an open discussion about systemic issues in academia, the related mitigating role of UHAND, and our own role as individual citizen scientists advocating for change. Being mindful that our scholars’ environment had impact on their work morale and performance, we stressed practicing routine self-care, engaging strategies for persistence and resilience, speaking up against injustices, and advocating for others as part of the professional experience. Acknowledging that the scholars felt overwhelmed, upset, and sometimes unsure of how to respond to the global and national crises, we shared professional resources, reminded scholars of our “open door” policy, and extended options to take days of rest and reflection. In sum, we applied a holistic approach in our mentorship, prioritizing fundamental training and professional development while including components of personal and population health and wellness. We recommend that other programs in their design combine such program features with rigorous mentored scientific research experiences to enable development of trainees as confident, well-rounded researchers with skills to conduct health disparities research and the resilience and wisdom to lead it.
